# Mycelial traits and GRSP in enhancing soil stability on cold region highway slopes: Comparative effects of three shrub species

**DOI:** 10.1371/journal.pone.0332483

**Published:** 2025-09-15

**Authors:** Yibo Li, Mingxin Zhou, Nan Li, Fei Ye, Li Yin, Hongtao Ma, Xingliang Xu

**Affiliations:** 1 Heilongjiang Institute of Construction Technology, Harbin, China; 2 Institute of Geographic Sciences and Natural Resources Research, CAS, Beijing, China; Rodale Institute, UNITED STATES OF AMERICA

## Abstract

In cold regions, the stability of highway slopes is crucial for infrastructure preservation, yet it remains highly vulnerable to soil erosion. This study investigated the role of mycelial traits in reinforcing soil aggregate stability by examining three shrub species—*Amorpha fruticosa* Linn. (AFL), *Lespedeza bicolor* Turcz. (LBT), and *Swida alba* Opiz. (SAO)—across two slope gradients (30° and 60°) in northeastern China. We measured water-stable aggregates, glomalin-related soil protein (GRSP) fractions, and mycelial traits. Results showed that AFL exhibited significantly greater aggregate stability than LBT and SAO, with its stability values 23.1–36.9% higher at the steep slope and 8.7–30.4% higher at the gentle slope. Strong correlations (r > 0.90) between EE-GRSP, mycelial traits, and aggregate stability explained up to 95.1% of the variance on gentle slopes, demonstrating a synergistic trait-based mechanism. However, slope gradient altered this coupling: GRSP efficacy diminished under steep slopes, leaving mycelial traits as the dominant driver of soil stability. These findings reveal a slope-dependent reallocation between physical scaffolding and biochemical adhesion, highlighting AFL and its mycelial traits as critical for slope stabilization in cold regions. The study provides a mechanistic basis for selecting shrub species in slope restoration and offers practical insights into erosion control under global change.

## 1. Introduction

Soil aggregate stability constitutes a fundamental process in slope ecosystems because it governs soil physical resilience against external disturbances such as rainfall-driven disintegration and particle detachment [1,2]. In sloping landscapes, topographic heterogeneity modifies water redistribution, erosion intensity, and structural reorganization, creating strong spatial variability in the mechanisms of soil stabilization [3,4]. These environmental filters can alter how biological and biochemical processes contribute to the maintenance of soil structure, potentially leading to nonlinear or context-dependent outcomes [5,6]. Despite substantial progress in soil aggregation research, current understanding of how slope gradients reshape the underlying stabilization mechanisms remains limited, leaving a critical gap in predicting soil structural resilience under heterogeneous terrains [7,8].

Several knowledge gaps constrain this field. First, previous studies have primarily addressed mycelial traits or GRSP fractions in isolation, rather than their coordinated contributions, which limits our understanding of how trait coupling generates aggregate stability [9,10]. Second, most evidence derives from flat or low-relief environments, leaving slope-induced heterogeneity in trait–function linkages largely unexplored, which restricts insights into topography-sensitive stabilization mechanisms [11]. Third, functional partitioning between structural scaffolding (hyphae) and biochemical binding (GRSP) has rarely been quantified across environmental gradients, constraining the ability to determine whether slope acts as a filter that reallocates the relative importance of these mechanisms [12]. Together, these gaps hinder a mechanistic framework that explains how plant-mediated belowground traits sustain soil physical resilience in slope ecosystems.

To address these limitations, we designed a factorial experiment combining three shrub species with contrasting ecological strategies and two slope gradients. The chosen species differ markedly in their investment strategies: AFL develops dense mycelial networks and secretes abundant GRSP, LBT exhibits moderate trait values, while SAO produces coarser hyphae but limited network extension. Such functional divergence provides a natural contrast for testing how morphological traits and protein secretion jointly regulate aggregate stability. By embedding these comparisons within slope contexts of 30° and 60°, our design captures both biological variation and topographic filtering, enabling a direct assessment of trait-mediated mechanisms across heterogeneous environments. This dual-axis framework offers unique potential to clarify how slope gradients restructure the coordination between structural and biochemical pathways of soil stabilization.

Given the contrasting ecological strategies of shrub species and the topographic heterogeneity imposed by slope gradients, we hypothesize that **(H1)** plant-mediated mycelial development and GRSP secretion jointly regulate soil aggregate stability through a trait-based soil–microbe interaction pathway. Specifically, species with greater mycelial biomass and coarser mycelial traits—such as AFL—are expected to enhance macroaggregate formation via increased physical enmeshment and elevated GRSP concentrations, particularly under lower slope conditions where moisture retention and root–microbe connectivity are more favorable. **(H2)** In this context, slope gradient is hypothesized to act as a modulatory factor that alters the strength and direction of belowground microbial feedbacks by influencing fungal proliferation, GRSP production, and the spatial architecture of soil particles. Moreover, the relative contributions of mycelial traits and GRSP fractions to aggregate stability are expected to exhibit slope-dependent variation, with mycelial morphological traits (e.g., volume and diameter) playing a dominant role in mechanically reinforcing aggregates, while GRSP functions as a biochemical binder with context-dependent efficacy. These interactions may reflect a topography-sensitive partitioning of microbial functional traits, forming an integrated mycelium–protein–structure continuum that determines soil physical resilience in sloped environments.

## 2. Materials and methods

### 2.1 Study area Introduction

The Hatong Expressway extends from Harbin to Tongjiang, crossing mountainous terrain, thus making slope stability a critical concern. To mitigate erosion and enhance stability, shrubs are planted along the expressway slopes. Our study focused on the section between Binxian and Fangzheng in northeastern China. This region experiences long, cold winters and short, warm summers, typical of a temperate continental monsoon climate. Precipitation is primarily concentrated during the summer months, posing challenges to the erosion resistance of expressway slopes in this region.

The selection of shrub species for this study followed established ecological and engineering standards. Through field investigations and by referencing relevant literature, the chosen species for investigation were *Amorpha fruticosa* Linn. (AFL), *Swida alba* Opiz. (SAO), and *Lespedeza bicolor* Turcz. (LBT). The study plots were characterized by the shrub attributes outlined in Table 1.

**Table 1:** Characteristics of important shrub species in the study area.

Species	Mean height (cm)	Mean coverage (%)	Canopy width (cm)	basal diameter (cm)
AFL	178.28 ± 3.76	96.5 ± 1.02	135.67 ± 9.66	1.87 ± 0.12
LBT	221.15 ± 5.66	92.4 ± 0.99	153.66 ± 11.23	2.25 ± 0.07
SAO	211.45 ± 2.66	92.7 ± 1.42	166.29 ± 10.57	1.92 ± 0.09

### 2.2 Soil sample collection

In the sampled area, surface soil samples of 0–20 cm were collected in four directions near each test shrub (AFL, LBT and SAO) under two slopes. Three replicate shrubs per species per slope were selected (n = 3 per species × 2 slopes = 18 total), following a balanced factorial design to ensure representation and statistical power. Soil samples of the same quality were collected at each point through multi-point sampling [13]. Non-soil impurities were removed, and the samples were appropriately labeled. The samples from each sampling point were immediately placed inside an ice pack and maintained at a temperature of 4 °C. They were then transported to the laboratory within 48 hours. After returning to the laboratory, the samples were thoroughly homogenized.

### 2.3 Determination of water stability of soil aggregates

This study employed the wet sieve method to measure the water stability of soil aggregates [14]. The procedure was as follows: A total of 1 kg of air-dried soil samples was collected from each sampling site. The samples were sieved through a series of mesh sizes (10 mm, 5 mm, 2 mm, 1 mm, 0.5 mm, 0.25 mm, and 0.1 mm) to separate soil aggregates by size. The mass of the soil retained on each sieve was recorded, and the percentage of the total soil mass was calculated for each size fraction. Based on the results of the dry sieving, 50 g of air-dried soil aggregates were mixed in proportion to the size distribution of the aggregates obtained from the previous step. This ensured that the sample accurately represented the soil’s aggregate structure. The prepared 50 g soil sample was evenly distributed over the top sieve of a wet sieve apparatus, with the mesh sizes arranged in descending order (10 mm, 5 mm, 2 mm, 1 mm, 0.5 mm, 0.25 mm, and 0.1 mm). The sieves were then slowly immersed in water until the sample on the top sieve was fully submerged. The apparatus was set to shake the sieves at a frequency of 35 oscillations per minute, with an amplitude of 4 cm, for 30 minutes. After the shaking was completed, the sieves were gradually lifted from the water, and the water-stable aggregates retained on each sieve were carefully collected. The collected aggregates were then dried in an oven at 105 °C until a constant weight was achieved. The dry mass of the aggregates from each sieve was measured, and the percentage of water-stable aggregates in each size fraction was calculated relative to the total sample mass. Using this method, we calculated the mean weight diameter (MWD) (Eq.1) and geometric mean diameter (GMD) (Eq.2) as follows [15]:


$$MWD=\sum_{i=1}^{n}x_{i}\omega_{i}$$ (Eq.1)

$$GMD=\exp[(\sum_{i=1}^{n}\omega_{i}\mathrm{lg}x_{i})/(\sum_{i=1}^{n}\omega_{i})]$$ (Eq.2)

In this formula, $x_{i}$ is the mean diameter of the soil size and the unit name is mm, and $\omega_{i}$ is the proportion of different soil granular aggregates to the total aggregates.

### 2.4 Mycelium extraction and determination of morphological characteristics

In the soil samples, this study extracted the external mycelium of the mycorrhizal fungi. The method in this study followed Malcová’s procedure for measuring mycelial length [16]. The specific operation steps are described as follows:

A 5 g soil sample was added to a high-speed grinder, then added 50 ml of sodium hexametaphosphate and stirred at 10,000 rpm for 30 seconds. The mixture was transferred to a 1000 ml beaker and stirred magnetically. After standing for 1 minute, the samples were screened with a 38 μm sieve, then transferred the fungi on the sieve with 250 ml of water to a 1000 ml beaker. This was followed by magnetic stirring at 900 rpm for 30 seconds. A 5 ml solution was then drawn from 1 cm below the liquid level and passed through a 0.45 μm microfilter, which was then placed on a slide. The tester repeated this process three times per sample, with three membranes per sample, and added three drops of trypan blue stain. After allowing the slides to air dry, a drop of lactic glycerol was added, and the slides were covered with coverslips. We magnified the mycelium in the filter membrane 10-fold using a BX-51 microscope (Olympus, Tokyo, Japan) and photographed them. We measured the total hyphal length, total hyphal surface area, total hyphal volume, and average hyphal diameter using the Root System Analyzer software (WinRhizo 2012b, Regent Instruments, Inc., Québec, Canada). Each soil sample was tested three times to obtain average mycelium trait values.

### 2.5 Determination of total and easily extractable glomalin-related soil protein (GRSP) content

The Bradford assay, based on Coomassie Brilliant Blue G-250 dye binding, is a widely used method for protein quantification [17]. Absorbance was read at 590 nm using a spectrophotometer (UV-1800, Shimadzu, Kyoto, Japan). Calculated the GRSP concentration using a known standard curve for protein concentration. GRSP was classified into two types based on extraction conditions: EE-GRSP, obtained using 20 mmol/L sodium citrate at pH 7.0 with 30-minute autoclaving, and T-GRSP, extracted using 50 mmol/L sodium citrate at pH 8.0 with 60-minute autoclaving.

Extraction and determination of EE-GRSP content: Following the established procedure described by Wright & Upadhyaya (1996), 0.5 g of soil was accurately weighed and transferred into a 10 mL centrifuge tube [18]. Next, 4 mL of sodium citrate solution (20 mmol/L, pH = 7.0) was added, and the tube was securely sealed. A blank sample was prepared by adding 4 ml of sodium citrate solution to a 10 ml centrifuge tube without soil. All samples were thoroughly mixed. The mixtures were thoroughly homogenized, after which the tube lids were briefly loosened, and the samples were autoclaved at 121 °C for 30 minutes. Once the autoclave pressure stabilized, the tubes were resealed and centrifuged at 4000 rpm for 6 minutes. The resulting supernatant was then collected, and Coomassie Brilliant Blue G-250 dye-binding method was performed to quantify the EE-GRSP content.

Extraction and determination of T-GRSP content: Following the procedure described by Wright & Upadhyaya (1996), 0.1 g of soil was precisely weighed and placed into a 10 mL centrifuge tube. Next, 4 mL of sodium citrate solution (50 mmol/L, pH = 8.0) was added, and the tube was tightly sealed. A blank sample was prepared by adding 4 ml of sodium citrate solution to a 10 ml centrifuge tube without soil. All samples were thoroughly mixed. Before placing the samples into the autoclave, the tester opened the centrifuge tube lids and autoclaved for 60 minutes at 121 °C. After the autoclave pressure dropped, the tester sealed the centrifuge tubes and centrifuged them at 4000 rpm for 6 minutes. The supernatant was collected and poured into a 50 ml centrifuge tube. This process was repeated for the soil samples until the supernatant no longer appeared red-brown, the typical color of GRSP. Finally, the supernatant in all 50 ml centrifuge tubes was mixed for measurement, and Coomassie Blue Staining was used to determine the T-GRSP content.

## 3. Statistical analysis

The normality and homogeneity of variance for all datasets were assessed using the Kolmogorov-Smirnov test and the Levene test, respectively. To explore the relationships between soil aggregates’ MWD and GMD with factors such as EE-GRSP, T-GRSP, total mycelial surface area, total volume, mean diameter, and total length, regression analysis was employed. The Tukey-Kramer HSD test along with one-way ANOVA was utilized to analyze MWD and GMD. Tukey-Kramer HSD inherently accounts for multiple comparisons by adjusting p-values across pairwise tests. Additionally, multifactor ANOVA was conducted to examine the effects of different plant species, slope gradients, and their interactions on various parameters.To explore the relationships between the measured variables, we generated a correlation matrix heatmap using Pearson’s correlation coefficient. The Random Forest algorithm was used to identify the importance of each feature for predicting MWD and GMD. OLS regression was performed to determine the coefficients of the features for predicting MWD and GMD. All data analyses were performed using SPSS version 22.0 (IBM Corp., Armonk, NY, USA). All data visualizations were generated using SigmaPlot version 12.5 (Systat Software Inc., San Jose, CA, USA).

## 4. Results

### 4.1 Soil Aggregate Stability and GRSP Concentrations under Different Plant Types and Slope Gradients

**Fig 1:**
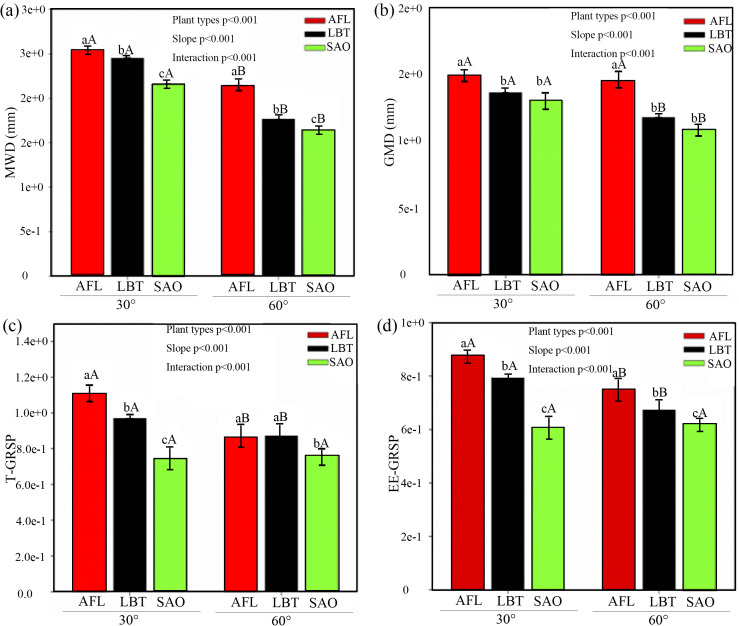
MWD (a) and GMD (b) at different slopes, T-GRSP (c) and EE-GRSP (d) at different slopes. Note: The figure presents the MWD, GMD, T-GRSP and EE-GRSP across different slope gradients for three plant types , expressed as mean ± SE. A general linear regression model (GLM) was used to examine the effect of plant types, slopes, and their interaction on the MWD, GMD, T-GRSP and EE-GRSP. The significance levels (p-values) were presented on the graphs. Different lowercase letters indicate statistically significant differences among plant types for the same soil slope , while different capital letters denote statistically significant differences within the same plant type across different soil slopes. A significant difference is indicated when P < 0.05. The unit of the T-GRSP and EE-GRSP is mg/g soil. AFL represents *Amorpha fruticosa* Linn., LBT represents *Lespedeza bicolor* Turcz., and SAO represents *Swida alba* Opiz.

At the 30° slope, AFL exhibited significantly higher values of MWD, GMD, T-GRSP, and EE-GRSP compared to both LBT and SAO, with LBT also significantly higher than SAO across all parameters (Fig 1a–1d). At the 60° slope, AFL consistently maintained significantly greater MWD and GMD than the other two species, whereas LBT showed significantly higher EE-GRSP than SAO; no significant differences in T-GRSP were observed among species (Fig 1a–1d). For each species, all four indices were significantly higher under the 30° slope than under the 60° slope, except for SAO, whose T-GRSP did not differ significantly between slope conditions (Fig 1a–1d).

### 4.2 Mycelial morphological traits under different plant types and slope gradients

**Fig 2:**
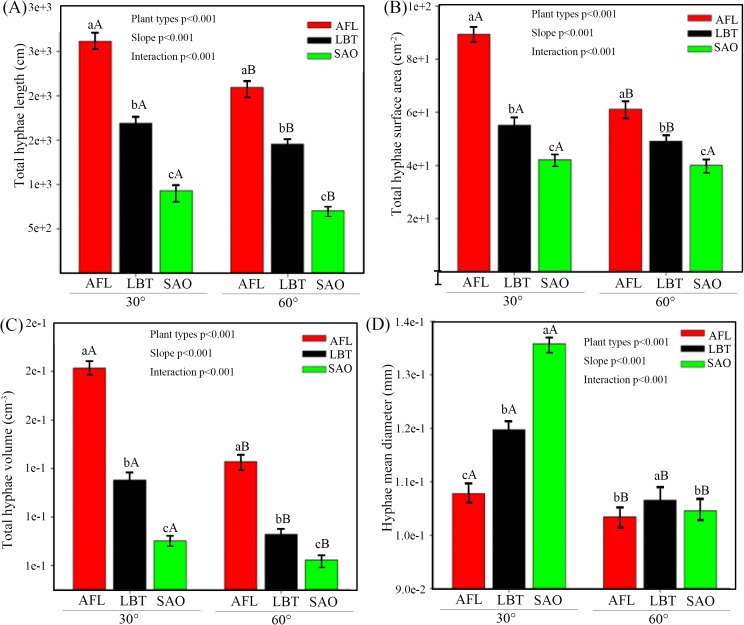
Hyphae characteristic under different slopes. Note: The significance levels (p-values) were presented on the graphs. Different lowercase letters indicate statistically significant differences among plant types for the same soil slope, while different capital letters denote statistically significant differences within the same plant type across different soil slopes. A significant difference is indicated when P < 0.05. The unit of the T-GRSP and EE-GRSP are mg/g soil. AFL represents *Amorpha fruticosa* Linn., LBT represents *Lespedeza bicolor* Turcz., and SAO represents *Swida alba* Opiz.

At the 30° slope, AFL exhibited significantly higher total hyphal length, surface area, and volume than LBT and SAO, with LBT significantly higher than SAO for all three traits; hyphal mean diameter of SAO was significantly higher than that of AFL and LBT. At the 60° slope, AFL maintained significantly higher total hyphal length, surface area, and volume than SAO, and significantly higher values than LBT in surface area and volume; hyphal mean diameter was significantly higher in SAO than in AFL and LBT. Intraspecific comparisons showed that all four mycelial traits were significantly higher at 30° than at 60° for AFL and LBT, whereas no significant slope-related differences were observed in SAO for surface area and mean diameter (Fig 2A–2D).

### 4.3 Correlations among Mycelial Traits, GRSP, and soil aggregate stability under different plant types and slope gradients

**Fig 3:**
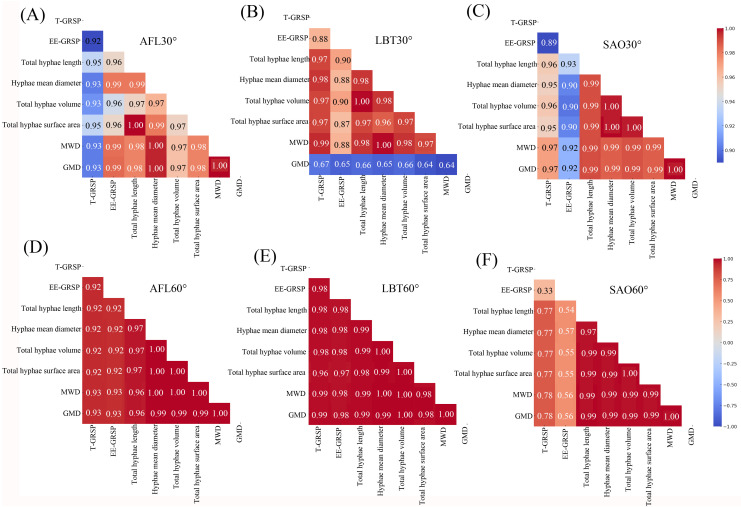
AFL heatmap analysis at 30 slope (A), LBT heatmap analysis at 30 slope (B), SAO heatmap analysis at 30 slope (C), AFL heatmap analysis at 60 slope (D), LBT heatmap analysis at 60 slope (E), SAO heatmap analysis at 60 slope (F). Note: The color intensity in the heatmap represents the strength of the correlation, with the legend on the right side displaying the corresponding correlation coefficient values. Strong positive correlations are shown in shades of red, while weaker correlations are shown in shades of blue. The closer the value is to 1 or -1, the stronger the linear relationship between the variables. The unit of the total hyphae length is cm, the unit of the hyphae mean diameter is mm, the unit of the total hyphae volume is cm^3^, the unit of the total hyphae surface area is cm^2^, the unit of the T-GRSP and EE-GRSP are mg/g soil, the unit of the MWD and GMD are mm. AFL represents *Amorpha fruticosa* Linn., LBT represents *Lespedeza bicolor* Turcz., and SAO represents *Swida alba* Opiz.

At the 30° slope, AFL exhibited significant positive correlations (r > 0.90) between EE-GRSP and all mycelial traits, and between total hyphal length and both MWD and GMD (Fig 3A). In LBT, EE-GRSP was significantly positively correlated with all mycelial traits, and both MWD and GMD were significantly positively correlated with mycelial volume and surface area (Fig 3B). In SAO, all mycelial traits showed significant positive correlations with MWD and GMD, while EE-GRSP was only positively correlated with total hyphal length and mean diameter (Fig 3C). At the 60° slope, AFL showed significant positive correlations among EE-GRSP, all mycelial traits, and aggregate stability metrics (MWD and GMD) (Fig 3D). In LBT, EE-GRSP was significantly positively correlated with total hyphal length, and all mycelial traits were positively correlated with MWD and GMD (Fig 3E). In SAO, mycelial traits were significantly positively correlated with each other and with MWD and GMD, while EE-GRSP showed weak or non-significant correlations with other traits (Fig 3F).

### 4.4 Predictors of soil aggregate stability under different slope gradients

**Fig 4:**
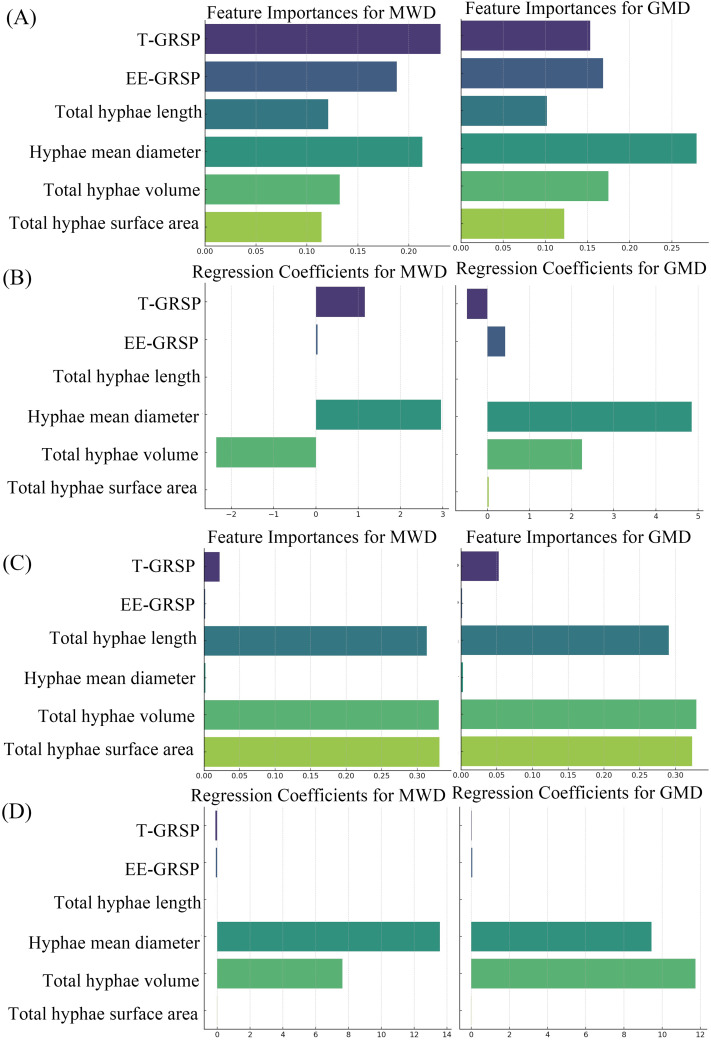
Feature Importance for MWD and GMD at 30 slope(A), Regression Coefficients for MWD and GMD at 30 slope(B). Feature Importance for MWD and GMD at 60 slope(C), Regression Coefficients for MWD and GMD at 60 slope(D).

At the 30° slope, hyphal mean diameter and total hyphal volume exhibited significantly higher feature importance for both MWD and GMD than other variables, as determined by the random forest model (Fig 4A), followed by EE-GRSP and total hyphal length. In the OLS regression, hyphal mean diameter and volume had the largest positive coefficients for both MWD and GMD, whereas T-GRSP showed significantly negative coefficients (Fig 4B). At the 60° slope, total hyphal length, volume, and surface area displayed the highest feature importance values across both MWD and GMD models, with hyphal mean diameter maintaining consistently high contributions (Fig 4C). Correspondingly, the OLS results indicated that hyphal mean diameter and volume had the strongest positive regression coefficients for both MWD and GMD, while T-GRSP and EE-GRSP contributed minimally or negatively (Fig 4D).

## 5. Discussion

### 5.1 Trait-mediated coordination of mycelial development and GRSP secretion determines aggregate stability

In support of our first hypothesis (H1), the results demonstrate that soil aggregate stability was strongly regulated by plant-mediated mycelial development and GRSP secretion, with clear interspecific differences modulated by slope gradients (Fig 1–3). Specifically, species investing more heavily in mycelial biomass and protein secretion, such as AFL, consistently enhanced MWD, GMD, and GRSP concentrations, thereby establishing a trait-based pathway through which structural resilience is maintained across heterogeneous slopes.

Among the studied species, AFL exhibited the strongest coupling between mycelial proliferation and soil structural enhancement, attributable to its extensive hyphal length, surface area, and volume, together with elevated EE-GRSP secretion. In contrast, SAO, despite exhibiting significantly larger hyphal diameters, contributed little to aggregate stability, suggesting that coarse but limited mycelial networks provide insufficient structural reinforcement [19,20]. LBT maintained intermediate values across traits and stability indices, reflecting a moderate functional strategy [21]. Such interspecific divergence aligns with earlier findings that plant-driven variation in mycelial morphology and GRSP concentration is a key determinant of aggregate stabilization [22,23]. These patterns emphasize that soil structural resilience is not dictated by mycelial size alone but by coordinated investment in both mycelial morphology and GRSP deposition [24,25].

The strong positive correlations between EE-GRSP, mycelial morphological traits, and aggregate stability, particularly in AFL and LBT (Fig 3A–E), underscore the integrative role of mycelial development in linking fungal physical structures with biochemical protein deposition. This evidence supports the view that EE-GRSP, as the more active fraction, exerts stronger binding effects than T-GRSP, consistent with reports that labile GRSP pools are more effective in promoting aggregate formation than recalcitrant pools [26,27]. Conversely, the weak or non-significant correlations observed in SAO (Fig 3C, 3F) indicate that limited mycelial biomass investment constrains the efficiency of this trait-based pathway. Together, these findings suggest a synergistic mechanism in which mycelial morphological traits provide physical enmeshment, while GRSP acts as a complementary biochemical binder, jointly enhancing soil structural stability [28,29].

Collectively, these results provide strong empirical support for H1 by demonstrating that interspecific variation in mycelial morphology and GRSP secretion jointly regulate soil aggregate stability through a trait-based pathway. The evidence chain indicates that species with acquisitive strategies, particularly AFL, enhance macroaggregate formation by simultaneously investing in mycelial development and GRSP deposition. From an applied perspective, these findings suggest that shrub species selection in slope ecosystems should prioritize those capable of sustaining extensive mycelial networks and higher GRSP concentrations to maximize soil structural resilience [30,31]. In the context of increasing erosion risks under global change, incorporating trait-based plant–soil interactions into slope restoration strategies could strengthen aggregate stability [32,33]. More broadly, these mechanisms highlight the necessity of integrating plant functional traits and soil biochemical pathways into predictive forest models under global change scenarios, where erosion, altered precipitation regimes, and vegetation shifts are expected to interactively shape soil structural resilience [34,35]. Future studies should extend these insights by adopting longer temporal scales, incorporating microbial functional diversity and enzymatic activities, and parameterizing trait–environment feedbacks in ecosystem models to better forecast soil stability under changing climates.

### 5.2 Slope gradient governs trait-mediated partitioning of mycelial and GRSP contributions to soil aggregation

In support of our second hypothesis (H2), our results demonstrate that slope gradient modulates the relative efficacy of mycelial structural traits versus GRSP fractions in soil aggregate stabilization. At the gentle slope (30°), hyphal mean diameter and volume were the strongest predictors of MWD and GMD, with EE-GRSP contributing positively, while T-GRSP exhibited negative associations; in contrast, at the steep slope (60°), structural mycelial traits remained crucial but GRSP’s contribution diminished or reversed (Fig 4A–4D). These responses paralleled slope-related declines in MWD, GMD, GRSP concentrations, and mycelial attributes (Fig 1a–1d; Fig 2A–2D), and the reshaping of correlation patterns in Fig 3A–3F.

Notably, AFL exhibited the most robust mycelial development and GRSP levels among species, under both slopes, resulting in higher structural stability; SAO, despite larger hyphal diameter, failed to bolster stability—indicating that network extension and surface provision, not thickness alone, are decisive (Fig 2D vs. Fig 1a–1d). These patterns correspond with trait-based findings showing that dense mycelial networks physically enmesh soil particles, whereas GRSP contributes biochemical binding where retention is feasible [36,37]. The steep-slope constraints likely attenuate GRSP retention via enhanced erosion and reduced moisture, limiting its adhesive efficacy [38,39].

Mechanistically, our findings unveil a slope-dependent trait partitioning: gentle slopes allow dual reinforcement (hyphae + EE-GRSP), while steep slopes rely predominantly on mycelial architecture [40,41]. This aligns with recent reports that structural traits dominate aggregate stability under disturbance or dryness, while GRSP is more effective under stable, moist conditions [42,43]. Such topography-sensitive trait integration supports theoretical frameworks in soil ecology suggesting functional trait hierarchies shift with environmental filters [44,45].

Together, these results validate H2, illustrating that slope gradients restructure functional trait contributions to soil aggregation. Our analysis (Fig 4) reinforces this mechanism by showing that hyphal mean diameter and volume consistently emerge as the dominant drivers of aggregate stability across slopes, while the contribution of GRSP fractions is slope-contingent—positive under gentle slopes but negligible or even negative under steep slopes—thereby confirming that slope gradient governs a functional reallocation between physical scaffolding and biochemical adhesion. From an applied standpoint, for slope stabilization, planting shrubs that foster extensive mycelial networks remains effective across gradients; gentler slopes may additionally benefit from practices enhancing EE-GRSP retention (e.g., moisture conservation, reduced disturbance) [46,47].

These findings highlight that slope-dependent trait partitioning represents a critical but often overlooked mechanism in forest ecosystems under global change, where shifts in precipitation and erosion regimes may redefine the balance between physical and biochemical drivers of soil stability [48,49]. Future studies should focus on identifying slope thresholds that trigger functional reorganization, comparing these dynamics across ecosystems with distinct topographies, and testing whether management interventions (e.g., terracing, mixed-species plantations) can modulate trait–environment interactions to sustain aggregate stability under intensifying climatic pressures [50,51].

## 6. Conclusion

This study demonstrates that soil aggregate stability is strongly regulated by plant functional traits related to mycelial development and GRSP secretion across slope gradients. Through trait-based comparisons and multi-variable integration, we identify three major insights: (i) Trait-mediated reinforcement of aggregates: Species investing more heavily in mycelial proliferation and EE-GRSP secretion, such as AFL, consistently enhanced soil structural stability, indicating that aggregate resilience arises from coordinated physical enmeshment and biochemical binding. (ii) Slope-dependent functional partitioning: Slope gradients modulated the relative efficacy of traits, with gentle slopes favoring dual reinforcement by hyphae and GRSP, while steep slopes constrained GRSP effectiveness, leaving structural mycelial attributes as the primary driver of stability. (iii) An integrated mycelium–protein–structure continuum: The evidence chain supports a hierarchical mechanism in which slope acts as an environmental filter, reconfiguring the balance between physical scaffolding and biochemical adhesion, thereby shaping soil resilience under heterogeneous topographic conditions.

From an ecological perspective, these findings underscore the importance of incorporating plant functional traits and belowground biochemical processes into forest management and slope restoration strategies. Shrub species capable of sustaining extensive mycelial networks and active GRSP pools, such as AFL, should be prioritized in reforestation programs, while management practices that enhance GRSP retention—such as moisture conservation and reduced disturbance—can further strengthen aggregate stability on gentler slopes.

Future research should extend these trait-based insights by employing longer temporal scales, integrating microbial functional diversity and enzymatic activities, and examining slope thresholds that trigger functional reorganization. In addition, parameterizing trait–environment feedbacks in ecosystem models will be critical for predicting soil structural resilience under global change, where altered precipitation and erosion regimes may redefine the balance between physical scaffolding and biochemical binding.

## Supporting information

S1 Data. (XLSX)
